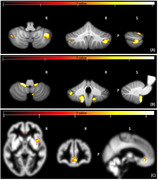# The Brain Tissue Properties Within the Cerebellum Play Role in Executive Functioning and Memory Performance in Healthy Older Adults

**DOI:** 10.1002/alz.094729

**Published:** 2025-01-09

**Authors:** Soodeh Moallemian, Chylinski Dapnée, Maxime Van Egroo, Narbutas Justinas, Eric Salmon, Maquet Pierre, Fabienne Collette, Vandewalle Gilles, Christophe Phillips, Christine Bastin

**Affiliations:** ^1^ Center for Molecular & Behavioral Neuroscience, Rutgers University–Newark, Newark, NJ USA; ^2^ GIGA‐CRC, University of Liège, Liège, Liège Belgium

## Abstract

**Background:**

Cognitive function alterations are a feature of the cognitive aging process. Additionally, aging is marked by macro‐ and micro‐structural changes in the brain, such as gray matter (GM) atrophy, iron accumulation, and demyelination. This study explores the association between cognitive function and cooccurrence of brain micro‐ and macro‐structural changes in healthy older adults.

**Method:**

One hundred and one participants (32% men, age range: 50‐69 years) were included in this study. All participants completed a cognitive assessment resulting in composite scores with mean = 0.00, s.d. = 0.99 for attention, executive functions, and memory. The preclinical Alzheimer’s cognitive composite (PACC5) was calculated for all participants (mean = 0.00, s.d. = 2.99). Quantitative magnetic resonance imaging (MRI) data were obtained using multiparametric mapping protocol. The association between cognitive composite scores and combinations of tissue properties was tested using multivariate generalized linear models (GLM) in Statistical Parametric Mapping (SPM) software.

**Result:**

Voxel‐wise multivariate GLM analyses revealed significant associations after family‐wise error rate correction between executive functions and the combination of macro‐ and micro‐structural changes within the cerebellum including right crus I and II, VII‐b (see Figure 1‐A). As illustrated in Figure 1, panels B and C, we also detected a correlation between memory and combined microstructural alterations in the in the left Cerebellum VIII‐b, as well as bilaterally within the cingulate gyrus and insula.

**Conclusion:**

These findings highlight the role of the cerebellum in cognition besides the complex relationship between cognition and brain micro‐ and macro‐structural properties in aging. As such, the involvement of the cerebellum in motor coordination and procedural memory may potentially influence executive functions